# A Mutation in INSR in a Child Presenting with Severe Acanthosis Nigricans

**DOI:** 10.4274/jcrpe.4577

**Published:** 2017-12-15

**Authors:** Hale Tuhan, Serdar Ceylaner, Özlem Nalbantoğlu, Sezer Acar, Ayhan Abacı, Ece Böber, Korcan Demir

**Affiliations:** 1 Dokuz Eylül University Faculty of Medicine, Department of Pediatric Endocrinology, İzmir, Turkey; 2 Intergen Genetics Centre, Ankara, Turkey; 3 University of Health Sciences, Dr. Behçet Uz Children Diseases and Surgery Training and Research Hospital, Department of Pediatric Endocrinology, İzmir, Turkey

**Keywords:** Rabson-Mendenhall syndrome, insulin resistance, INSR

## Abstract

Rabson-Mendenhall syndrome (RMS) is an autosomal recessive disorder due to mutations in the insulin receptor gene (INSR) which is mapped to 19p13.2. RMS is characterized by acanthosis nigricans, generalized lanugo, tooth and nail dysplasia, high nasal bridge, and growth retardation. A 5-year-old female patient was referred due to acanthosis nigricans and generalized lanugo. On her physical examination, severe acanthosis nigricans of the neck, axillae, the external genitalia and antecubital regions, generalized lanugo, mildly decreased subcutaneous fat, dysmorphic facial features, and polydactyly on her left hand were noted. Insulin resistance and impaired glucose tolerance were found. Sequence analysis of the INSR in the patient revealed c.3529+5G>A mutation in homozygous state. RMS should be suspected in a patient with characteristic physical features and insulin resistance.

**What is already known on this topic?**

Mutations in INSR lead to a wide spectrum of insulin resistance syndromes ranging from leprechaunism to type A insulin resistance. Rabson-Mendenhall syndrome (RMS) is an intermediate form of insulin resistance syndrome since the function of insulin receptor is only moderately reduced.

**What this study adds?**

To the best of our knowledge, we report the first case with RMS from Turkey diagnosed at molecular level. Sequencing of INRS revealed a novel homozygous mutation.

## INTRODUCTION

The human insulin receptor (IR) consists of two extracellular α subunits and two transmembrane intracellular β subunits. Insulin binds to α subunit and activates β subunit autophosphorylation and kinase activity, which is essential for transmembrane signaling of glucose transport. The α and β subunits of the IR are encoded by a single gene (INSR) which is mapped on the short arm of chromosome 19 (1).

Mutations of INSR lead to a wide spectrum of inherited insulin resistance syndromes ranging from leprechaunism (Donohue syndrome, autosomal recessive), which occurs in infancy and results in death, to type A insulin resistance (autosomal dominant), which leads to mild clinical symptoms after puberty. The severity of Rabson-Mendenhall syndrome (RMS) (autosomal recessive) is intermediate between the two aforementioned types (2). In RMS, the function of IR is less severely reduced, while little or no residual IR function is found in leprechaunism (3).

The loss of IR function results in various metabolic and growth defects. Metabolic defects in RMS are characterized by fasting hypoglycemia, postprandial hyperglycemia, later refractory hyperglycemia, extreme hyperinsulinemia, and late ketoacidosis. Affected patients also have postnatal growth restriction as well as impaired muscle and adipose tissue development due to the defective mitogenic action of insulin (3). Clinical findings of RMS include acanthosis nigricans, generalized lanugo, tooth and nail dysplasia, high nasal bridge, growth retardation, and hyperextensible joints (4). It is a rare genetic disorder, and in our country, no case with RMS diagnosed at molecular level has been reported to date.

## CASE REPORT

A 5-year-old female patient was referred due to acanthosis nigricans and generalized lanugo which developed in the last two years. According to the past medical history, she was born by cesarean section after an uneventful pregnancy with a birth weight of 3500 g and length of 49 cm. She was the first child of first-degree consanguineous parents. Her developmental milestones were normal. There is no family history of any remarkable medical problems. She had a healthy sibling who did not have any dysmorphic features.

**Figure 1:**
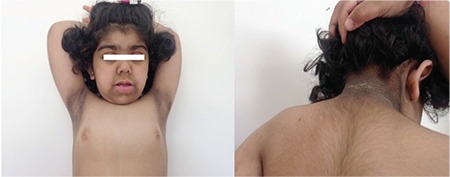
Dysmorphic features of the patient and acanthosis nigricans on her neck

Her weight was 18.7 kg [standard deviation (SD) score -0.08], height 98.7 cm (SD score -2.6), and body mass index 19.2 kg/m^2^ (SD score 1.94). Severe acanthosis nigricans of the neck, axillae, the external genitalia and antecubital regions, generalized lanugo, mildly decreased subcutaneous fat, coarse face, large ears, high nasal bridge, upturned nose, abnormalities of the teeth, gingival hyperplasia, and polydactyly in her left hand were noted (Figure 1). Complete blood count, liver and renal function tests, electrolytes, lipid profile, fasting and postprandial glucose levels, glycosylated hemoglobin (HbA1c), thyroid function tests, insulin-like growth factor (IGF) 1, and serum IGF binding protein-3 levels were normal, while fasting insulin was extremely high (Table 1). After an overnight fast, an oral glucose tolerance test (OGTT) (1.75 g/kg) was performed and impaired glucose tolerance was detected. Bone age was consistent with 4 years according to the Greulich-Pyle atlas. Healthy eating and lifestyle changes were recommended to the patient for impaired glucose tolerance. Clinical features and metabolic status were found to be unchanged after one year of follow-up.

### Molecular Studies

**Figure 2:**
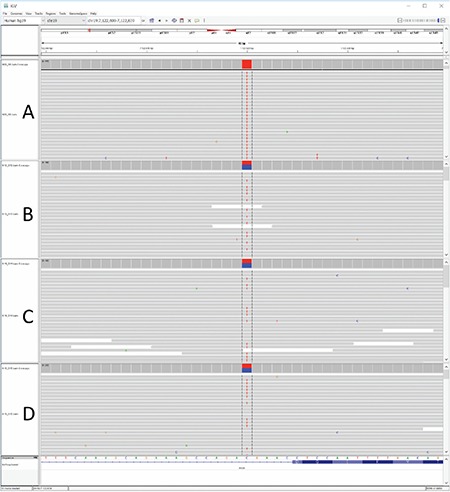
Homozygous (A, patient) and heterozygous (B, sibling; C, father; D, mother) INSR mutations identified in the family members

After getting informed consent from the parents, DNA was extracted from peripheral leukocytes using standard methods. All exons and flanking intron regions of the INSR (NM_000208.3) were sequenced and a homozygous mutation was found: c.3529+5G>A (IVS19+5G>A) (Figure 2). This impact of variant was analyzed by using Human Splicing Finder V3 and Mutation Taster and both predicted this variant to be damaging. Our literature search yielded a heterozygote variant in a case with Donohue syndrome with compound heterozygote genotype (p.Arg1027* in exon 17 and c.3529+5G>A) that was recently reported in a congress session (5). There is no functional analysis data in the literature. Frequency of this variant was 4 in 121408 in EXAC database. This variant was also present in dbSNP database with rs764083259 code. DANN score is 0.8688.

Screening for the relevant mutation was performed in family members. The parents and the 3-year-old sibling were heterozygous for the same mutation (Figure 2).

## DISCUSSION

RMS was first described in 1956 by Rabson and Mendenhall. Acanthosis nigricans, hypertrichosis, skin and dental abnormalities, phallic enlargement, prognathism, abdominal distension, pineal hyperplasia, coarse facies, insulin-resistant diabetes, growth retardation, lack of subcutaneous fat, lichenified skin, and fissuring of the tongue were reported to be the clinical symptoms of RMS (6,7). In our patient, since the age of 3 years, several symptoms of this condition (acanthosis nigricans, hypertrichosis, teeth abnormalities, gingival hyperplasia, and dysmorphic face) were present. Analysis of the INSR yielded a novel homozygous mutation.

Variant distributions given in UniProt database in both RMS and leprechaunism do not present a clear cut domain related distribution. Clinical variations seem mostly related with severity of functional effect of the mutation. The present mutation was first reported as a component of a compound heterozygous mutation found in a case with leprechaunism. The severe clinical picture was most probably due to the frame-shift mutation of the second allele of that patient (5). It is not possible to reach a similar conclusion for our patient without a functional analysis in such a splice site mutation at +5 position. However, in silico analyses indicate that the variant is damaging and that there was no other alteration in INSR.

Patients with RMS can develop fasting hypoglycemia, postprandial hyperglycemia, and ketoacidosis (8). In our patient, according to the OGTT, impaired glucose tolerance was detected. Since serum glucose level was not too high at 2-h OGTT, only diet and lifestyle changes were recommended as treatment.

Thakker (2) have reported 11 patients with RMS and type A insulin resistance who were diagnosed with diabetes at presentation and treated with high-dose insulin and insulin-sensitizing drugs. However, it has been also reported that, despite use of high doses of insulin or oral drugs like metformin and glitazones, poor glycemic control persisted in most patients with RMS (2). There is still no adequate treatment for RMS. In some studies, recombinant human IGF-1 has been shown to reduce fasting glucose, fasting insulin, C-peptide, and proinsulin levels and to improve glycemic control (9). However, these were short-term studies.

Another treatment approach for RMS is recombinant methionyl human leptin therapy (metreleptin). The beneficial effects of metreleptin on severe insulin resistance due to lipodystrophy syndromes and leptin deficiency are well known. Cochran et al (10) have demonstrated a significant reduction in blood glucose over 10 months in two patients with RMS. Brown et al (11) also demonstrated a 1.7% reduction in HbA1c with metreleptin treatment for 12 months in five patients with RMS. These authors stated that metreleptin could be an option for the treatment of RMS, but that additional therapies were needed to improve glycemic control (11). As another recent approach, vildagliptin, an oral anti-diabetic agent of the new dipeptidyl peptidase-4 inhibitor class of drugs, was reported by Moreira et al (12) to be an option for treatment of insulin resistance in cases with RMS. In their patient, vildagliptin was used as part of combination therapy which included metformin, pioglitazone, and acarbose (12). It is clear that long-term studies are needed to fully assess the effect of vildagliptin on insulin resistance syndromes. Since our patient did not need any medication in the follow-up, we have no experience with the above compounds.

In conclusion, this report describes a homozygous INSR mutation resulting in a clinical picture of insulin resistance and dysmorphic features of RMS and emphasizes the importance of screening the INSR in patients with insulin resistance and dysmorphic features. At the moment, there is no definitive treatment for RMS and new treatment approaches are needed.

## Figures and Tables

**Table 1:**
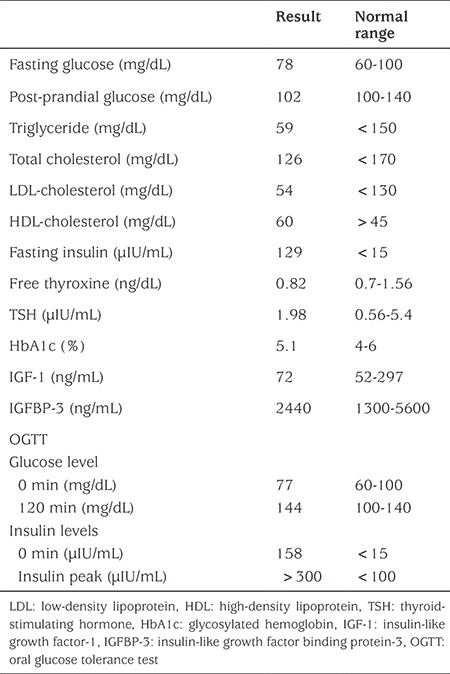
Laboratory findings of the patient
